# Assessment of using Google Trends for real-time monitoring of infectious disease outbreaks: a measles case study

**DOI:** 10.1038/s41598-024-60120-8

**Published:** 2024-04-24

**Authors:** Dawei Wang, John Cameron Lang, Yao-Hsuan Chen

**Affiliations:** 1 Health Economic and Decision Sciences, Merck & Co., Inc., Rahway, NJ USA; 2 Health Economic and Decision Sciences, Merck Canada Inc., Kirkland, QC Canada; 3 Health Economic and Decision Sciences, MSD (UK) Limited, London, UK

**Keywords:** Measles, Infectious disease, Disease outbreak, Epidemiology, Disease surveillance, Google Trends, Epidemiology, Epidemiology, Outcomes research

## Abstract

Measles remains a significant threat to children worldwide despite the availability of effective vaccines. The COVID-19 pandemic exacerbated the situation by leading to the postponement of supplementary measles immunization activities. Along with this postponement, measles surveillance also deteriorated, with the lowest number of submitted specimens in over a decade. In this study, we focus on measles as a challenging case study due to its high vaccination coverage, which leads to smaller outbreaks and potentially weaker signals on Google Trends. Our research aimed to explore the feasibility of using Google Trends for real-time monitoring of infectious disease outbreaks. We evaluated the correlation between Google Trends searches and clinical case data using the Pearson correlation coefficient and Spearman’s rank correlation coefficient across 30 European countries and Japan. The results revealed that Google Trends was most suitable for monitoring acute disease outbreaks at the regional level in high-income countries, even when there are only a few weekly cases. For example, from 2017 to 2019, the Pearson correlation coefficient was 0.86 (*p*-value< 0.05) at the prefecture level for Okinawa, Japan, versus 0.33 (*p*-value< 0.05) at the national level for Japan. Furthermore, we found that the Pearson correlation coefficient may be more suitable than Spearman’s rank correlation coefficient for evaluating the correlations between Google Trends search data and clinical case data. This study highlighted the potential of utilizing Google Trends as a valuable tool for timely public health interventions to respond to infectious disease outbreaks, even in the context of diseases with high vaccine coverage.

## Introduction

Measles virus is one of the most infectious viruses on the planet^1^ and a leading cause of death and disability-adjusted life-years lost^2^. With a basic reproduction number (i.e., the number of cases directly generated from one case in a a susceptible population) of 12–18^1^, its transmissibility far exceeds other diseases, including SARS-CoV-2, which has a reproduction number of 2.5–3.5^3^ and its Omicron variant, which has a reproduction number of 8.2^4^. About 75–90% of susceptible household contacts develop the disease^5–7^. Before the introduction of measles vaccines, 95–98% of children were infected by the measles virus by age 18^8–11^.

Sixty years after effective vaccines were licensed in 1963, measles continues to cause death and diseases in children worldwide. In 2018, the World Health Organization (WHO) reported more than 140,000 measles deaths globally, mostly among children under the age of 5^12^. Complications from measles can occur in almost every organ^13^. Measles infection can also diminish previously acquired immune memory, potentially leaving individuals at risk for reinfection by previously acquired pathogens^14^. Studies during the 1970s and 1980s revealed that measles case-fatality rates ranged from 3 to 34%^15–17^ in low- and middle-income countries (LMICs), 10–20 times higher than high-income countries^13^. Although measles vaccines are highly effective with an efficacy of 97%^18^, outbreaks still occur in places with low vaccination coverage rates. Significant, yet inconsistent, progress has been made in measles vaccination since 2000. From 2000 to 2016, measles cases worldwide decreased from 145 to 18 cases per million, after which they increased again to 120 cases per million in 2019^19^.

Although measles cases did decrease during the COVID-19 pandemic (to 22 cases per million in 2020)^19^, millions more children were susceptible to measles at the end of 2020 than in 2019. Specifically, 22.3 million children among 194 WHO member states and at least 93 million persons in 23 countries did not receive measles-containing vaccines (MCVs) because of COVID-19-related postponement of measles supplementary immunization activities (SIAs) for 2020^19^. Measles surveillance also deteriorated during COVID-19^19^. In 2020, the number of measles specimens submitted was the lowest in over a decade. Many countries did not report, and few countries (32%) achieved the measles surveillance sensitivity indicator (i.e., the proportion of cases that have an imported source)^20^.

Increased population susceptibility and suboptimal measles surveillance portend an immediate elevated risk for measles transmission and outbreaks, threatening the already fragile progress toward regional elimination goals^19^. Furthermore, measles cases were not only in low-vaccination LMICs but also in high-vaccination high-income countries. In 2018, 47 of 53 Member States of the WHO European Region reported over 84,000 confirmed measles cases. Cases rose by 300% during the first 3 months of 2019 compared with the same period in 2018^21^. Although endemic measles was declared “eliminated” from the United States^22^, more than 1200 confirmed cases were reported in 31 states in 2019^23^.

The deteriorated surveillance over an increased susceptible population of one of the most infectious viruses highlights the value of real-time surveillance systems for measles. The WHO has recommended the Moving Epidemic Method (MEM) as a tool for assessing the severity of epidemics^24,25^. We previously applied the MEM to Google Trends search data for respiratory syncytial virus (RSV) to demonstrate the feasibility of using Google Trends as a data source for real-time monitoring of RSV outbreaks^26^. This approach complements existing surveillance systems to monitor disease outbreaks in real-time, especially in countries with limited or no sentinel network surveillance. An important step in validating this surveillance approach is to obtain both Google Trends search data and clinical case data to verify that these data are highly correlated and result in equivalent estimates for outbreak thresholds. In this study, we aim to explore the feasibility of extending this surveillance approach to other diseases, using measles as a worked example. Compared to previous work for RSV, which has no widespread immunization program, 81% and 71% of children had received 1 and 2 doses of measles-containing vaccines respectively in 183 WHO member states by the end of 2021^27^. This high vaccination coverage could lead to much smaller outbreaks and potentially much weaker signals reflected on Google Trends. Consequently, other studies have found high correlation between monthly clinical case and Google Trends data over measles by summing up 3 countries’ Google Trends signals and cases for Italy, France, Germany, and Romania during 2013–2018 due to each country’s weak Google Trends signal^28,29^.

This study aimed to provide guidance for evaluating whether Google Trends can be applied to monitoring other diseases, such as measles. If Google Trends search data is found to be highly correlated with disease clinical case data in the context of a highly-vaccinated disease like measles, then previously published methods can be adapted to establish a pseudo-surveillance system for measles. We developed insights into what disease outbreak patterns are captured by Google Trends at both country and regional levels, how to better utilize these data, and limitations of using Google Trends to monitor disease outbreaks. We also share insights of which similarity measurements may be more suitable for this particular task. Popular performance measurements are adopted with further justification in this application area. However, those widely used performance measurements could lead to dramatically different conclusions^30,31^.

## Methods

Correlation analysis of measles between Google Trends search data and clinical case data was performed to evaluate if Google Trends search data are highly correlated with clinical case data, even for highly vaccinated diseases like measles. If so, then the same methods from the previous study^26^ can be easily adapted to other diseases to establish the pseudo-surveillance system. The analysis was performed at the country level across 29 EU/EEA Member States and the UK. Japan and Germany were investigated at the regional level. With limited clinical case data, only Google Trends search data of the top 10 countries with the largest number of measles cases from October 2022 to March 2023 were evaluated.

### Data

Monthly measles clinical case data for 29 EU/EEA Member States and the UK from 2016/04 to 2020/02 were collected from the European Centre for Disease Prevention and Control (ECDC) monthly measles and rubella monitoring reports^32^. Empty entries were filled with the floor of the average for previous and next months. Japan and Germany were selected for further investigation at the regional level, as the weekly case reports of those two countries at regional level were available. Weekly measles clinical case data in Germany from 2017 to 2019 were obtained from SurvStat database provided by Robert Koch Institut (RKI)^33^. Weekly measles clinical case data for Japan from 2017 to 2019 were gathered from the National Institute of Infectious Diseases (NIID)^34^.

Google Trends^35^ search data reflects how a specific search interest varies for a region over time, ranging from 100 to 0%, scaled by the highest search number that a specific search interest ever generated within the chosen time period. Weekly or monthly data points are extracted if the chosen time period is shorter or longer than 5 years, respectively. The keyword “麻疹”, in Japanese was used for Japan, and “Measles” in boèth English, as well as translations into the first language of each European country using Google Translate, were used. The keyword “Measles”, in English, was used for the top 10 countries with the largest number of measles cases from October 2022 to March 2023.

### Measurement

Both Pearson’s correlation coefficient (PCC) and Spearman’s correlation coefficient (SRCC) were calculated between Google Trends and clinical case data. PCC measures the linear correlation between two sets of data, while SRCC measures the rank correlation (i.e., the statistical dependence between the rankings of two variables). Both range from − 1 to 1, with 1 indicating perfect correlation, 0 indicating no correlation, and − 1 indicating perfect anti-correlation. PCC does not imply significance of SRCC (and vice versa)^36^. Results of both estimators with the statistical significance levels of 0.05 and 0.01 were listed, as both statistics have been used in previous studies^26,29^. The Python library package SciPy^37^, was used to perform the correlation analyses.

## Results

### Outbreaks captured in Google Trends for high-income countries

**Figure 1:**
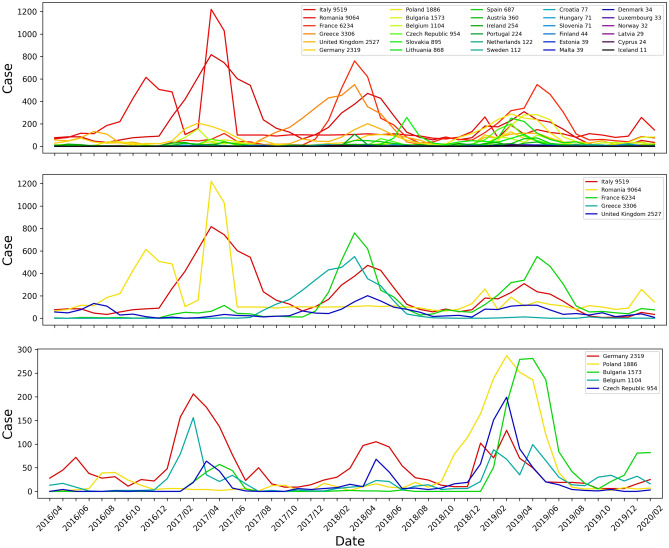
The monthly number of measles cases for 29 EU/EEA member states and the UK from 2016/04 to 2020/02. Number of total cases are indicated after the country name in the legend. Plots of 10 countries with the most number of cases are shown in lower figures in various scales to show the outbreak trends.

**Figure 2:**
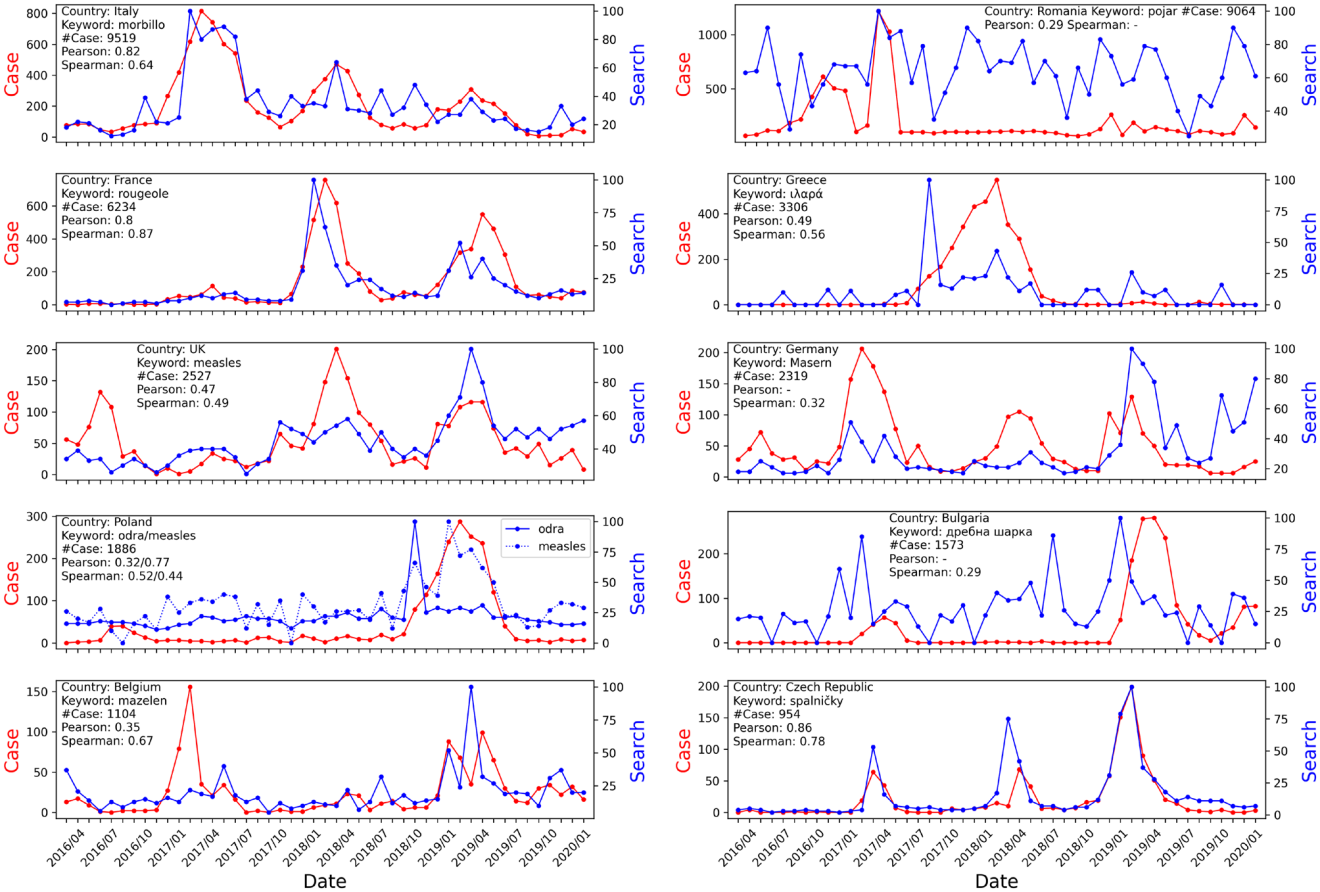
Correlation between monthly Google Trends and clinical case data of top 10 EU/EEA member states and the UK by month from 2016/04 to 2020/02.

The monthly number of measles cases for all 29 EU/EEA member states and the UK from 2016/04 to 2020/02 is shown in Fig. 1. For illustration purposes, among 30 countries, the top 10 countries ranked by number of total cases showed clear acute outbreak patterns in Fig. 1. Correlations between monthly Google Trends search and clinical case data of the top 10 member states and the UK by month from 2016/04 to 2020/02 are shown in Fig. 2. The results for all countries are listed in Table 1. Countries with blank results are due to: (1) The measurement is not statistically significant (*p*-value$\ge$0.05); (2) No search activities for the specified keyword were captured on Google Trends data during the selected time period. Google Trends with keywords in each country’s official language usually resulted in a higher correlation with clinical case data compared to keywords in English. A search with keywords combined in multiple languages does not necessarily result in a higher correlation.

Measles outbreaks were not captured on Google Trends for LMICs. The top 10 countries with the largest number of measles cases ranged from 68,473 (India) to 1769 (Nigeria) from October 2022 to March 2023^38^ were investigated. Only India showed clear patterns on Google Trends.

**Table 1:** Correlation between monthly Google Trends and clinical case data for 29 EU/EEA member states and the UK from 2016/04 to 2020/02.

		Official			English		Official + English	
Country	#Case	Keyword	Pearson	Spearman	Pearson	Spearman	Pearson	Spearman
Italy	9519	morbillo	0.82	0.64	0.45	0.38	0.82	0.65
Romania	9064	pojar	0.29*	–	–	–	–	–
France	6234	rougeole	0.8	0.87	0.31*	0.49	0.8	0.87
Greece	3306	ı$\uplambda \upalpha \uprho \acute{\upalpha }$	0.49	0.56	–	0.32*	0.29*	0.58
United Kingdom	2527	measles	0.47	0.49	0.47	0.49	0.47	0.49
Germany	2319	Masern	–	0.32*	–	–	–	0.3*
Poland	1886	odra	0.32*	0.52	0.77	0.44	0.34*	0.54
Bulgaria	1573	дребна шарка	–	0.29*	–	0.41	–	0.37
Belgium	1104	mazelen	0.35*	0.67	–	0.51	0.39	0.73
Czech Republic	954	spalničky	0.86	0.78	0.67	0.41	0.87	0.7
Slovakia	895	osýpky	0.76	0.57	0.32*	0.34*	0.74	0.6
Lithuania	868	tymų	0.9	0.38	0.53	0.4	0.93	0.47
Spain	687	sarampión	–	0.3*	0.54	0.37	–	0.36*
Austria	360	measles	0.58	0.31*	0.58	0.31*	0.58	0.31*
Ireland	254	an bhruitíneach	–	–	0.64	0.73	0.65	0.71
Portugal	224	sarampo	0.63	0.64	0.31*	0.46	0.62	0.61
Netherlands	122	mazelen	–	0.56	0.41	0.29*	–	0.52
Sweden	112	mässling	0.82	0.43	0.49	–	0.82	0.4
Croatia	77	ospice	0.71	0.42	0.29*	–	0.69	0.42
Hungary	71	kanyaró	–	0.38	–	–	–	0.37*
Slovenia	71	ošpice	0.71	0.41	–	–	0.69	0.36*
Finland	44	tuhkarokko	0.75	0.67	0.53	0.48	0.75	0.7
Estonia	39	leetrid	0.74	0.62	0.46	0.52	0.7	0.51
Malta	39	${\hbar }$osba	–	–	0.32*	–	0.32*	–
Denmark	34	mæslinger	0.72	0.44	0.54	0.38	0.73	0.51
Luxembourg	33	Maselen	–	–	0.52	–	0.52	–
Norway	32	meslinger	0.78	0.56	0.43	0.29*	0.78	0.55
Latvia	29	masalām	–	–	–	–	–	–
Cyprus	24	ı$\uplambda \upalpha \uprho \acute{\upalpha }$	–	–	–	–	–	–
Iceland	11	mislingum	–	–	0.56	–	–	–

Results of both Spearman’s rank and Pearson correlation coefficient measurements were provided. Keyword of “measles” in official, English, and combined (official+English) languages for each country were evaluated. Results with *p*-value$<0.01$ were listed. Results with *p*-value$<0.05$ were marked with ‘*’ at the end.

### Accurate acute outbreaks captured in Google Trends at regional level

**Figure 3:**
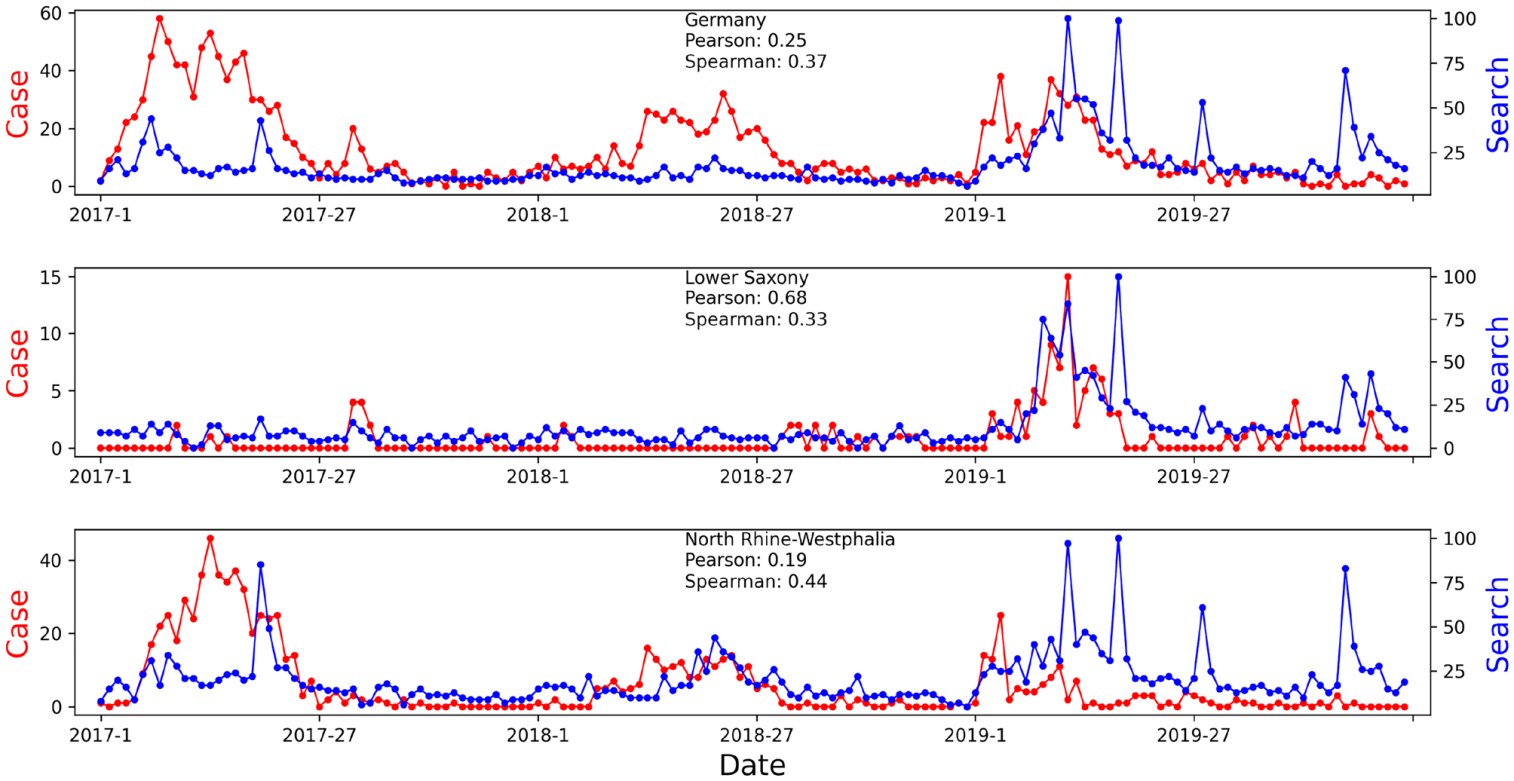
Correlation between weekly Google Trends search and clinical case data of Germany and regions with most Google searches and cases between 2017 to 2019.

**Figure 4:**
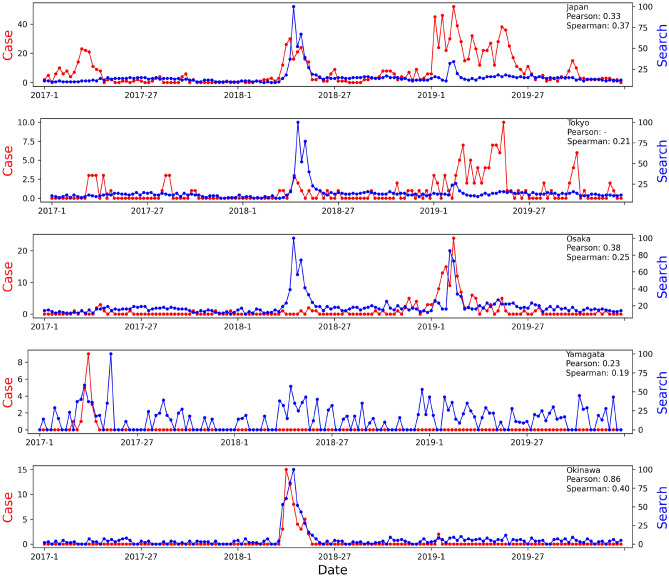
Correlation between weekly Google Trends search and clinical case data of Japan and regions with most cases between 2017 to 2019.

High correlations were found between weekly Google Trends search and clinical case data. Germany and Japan were investigated at regional level. For Germany, low correlations for either the Pearson correlation coefficient (PCC) (0.25) or the Spearman’s rank correlation coefficient (SRCC) (0.37) measurements were observed at the country level, as shown in Fig. 3. At the regional level, two states were selected for illustration purposes. Lower Saxony was selected because it had the highest Google Search volumes compared to all other states. North Rhine-Westphalia was selected because it had the highest number of cases from 2017 to 2019. At the country level, the outbreak in 2018 was completely missed on Google Trends. However, it was well captured on Google Trends in regions where the outbreak occurred (e.g., North Rhine-Westphalia). Regions (e.g., Lower Saxony) without any outbreak in 2018 showed no activity on Google Trends as well. Similar observations were found in Japan. At the country level, both low correlations for PCC (0.33) and SRCC (0.37) measurements were observed from 2016 to 2019 as shown in Fig. 4. In 2017, the outbreak was not captured on Google Trends for at the country level, but it was captured on Google Trends of Yamagata, where the outbreak occurred. In 2018, although Google Trends search and clinical case data aligned well, Google Trends of big cities (e.g., Tokyo, Kyoto) also captured search volume spikes, where no outbreak happened. The outbreak was mainly in Okinawa. In 2019, the amplitude of Google Trends signals was far lower than clinical case data. This is because several cases happened in multiple regions, adding up to a high number of weekly cases at the country level. Acute outbreaks (sudden large numbers of cases within a short period of time) were captured on Google Trends in Osaka. However, there were few cases circulating around during a long period of time in Tokyo, which did not trigger a high search volume spike pattern on Google Trends.

### The Pearson correlation coefficient more suitable than the Spearman’s rank correlation coefficient

The Pearson correlation coefficient (PCC) seems to be more suitable than Spearman’s rank correlation coefficient (SRCC) estimation for this task. For example, for Poland, as shown in Fig. 2, using the keyword in English for Google Trends resulted in a pattern more similar to the clinical case data, leading to a higher PCC (0.77 vs. 0.32) and a lower SRCC (0.44 vs. 0.52) compared to using the “odra” keyword in Polish. For Belgium, the first spike in clinical data was completely missed in Google Trends, resulting in a low PCC (0.35), but a high SRCC (0.67). In Japan, as shown in Fig. 4, Okinawa showed perfect correlation between Google Trends search and clinical case data. However, the SRCC only yielded a low value of 0.40, while the PCC showed 0.86.

## Discussion

Google Trends can complement existing surveillance systems for monitoring disease outbreaks in real-time. High correlations between Google Trends search and clinical case data were observed for measles. It is most suitable to monitor acute disease outbreaks at the regional level in high-income countries. Although these high-income countries usually have high-quality weekly case reports, we observed that weekly reports may be delayed for several weeks due to various reasons. On the other hand, Google Trends is able to provide weekly trends in real-time. It can also be used as a supplemental surveillance system for countries with limited sentinel network coverage.

Occasionally, a single keyword such as “measles” in the first language could be sufficient for identifying the clear outbreak patterns for measles on Google Trends in most countries. Adding the keyword “measles” in English may result in noisier data, which could lower the accuracy of monitoring outbreaks using Google Trends.

When estimating correlations between Google Trends search and clinical case data, the Pearson correlation coefficient seems to be more suitable than Spearman’s rank correlation coefficient for this particular task.

Previous studies have only investigated the correlations between clinical case data and Google Trends search data for measles at the country level^28,29,39^. For example, due to the weak signal from Google Trends data, Samaras and colleagues aggregated Google Trends data from three countries to evaluate the correlation with clinical case data^29^. In contrast, we evaluated correlations at the regional level and found that correlations between clinical case data and Google Trends were stronger at the regional level than the national level. Using this approach in developing a pseudo-surveillance system has greater potential to localize disease outbreaks.

### Limitations

There are also limitations to using Google Trends to monitor disease outbreaks. At the country level, Google Trends does not work well in LMICs. This may be due to poor Internet infrastructure limiting Internet access, low education levels, or low healthcare coverages, limiting knowledge-seeking behaviors. In high-income countries, compared to acute outbreaks, Google Trends cannot capture prolonged outbreaks with very few cases (<10 cases/week) circulating around all the time, such as the outbreaks in Tokyo in 2019 shown in Fig. 4. This may be due to the disease being around for too long but not widespread, causing people not to worry to continue to search. Also, local signals on Google Trends may not necessarily mean local outbreaks, such as the spikes on Google Trends of Tokyo and Osaka in 2018. This may be due to searches in big cities are coming from people like news staff, healthcare officials, or researchers, whose searches are not related to local outbreaks only. However, big cities usually have alternative existing surveillance systems to confirm whether there is a local outbreak. Google Trends data are sensitive to the selection of keywords. In this paper, we’ve only used one keyword to identify trends for our preliminary investigation, which could be more prone to false alerts triggered from news that may not relate to disease outbreaks.

## Conclusion

This paper investigated the adaptation and feasibility of monitoring disease outbreaks using Google Trends data in real-time, especially for countries and diseases with limited or no sentinel network surveillance system. Using measles as an extreme case, which was much less widespread due to high vaccination coverage rates and early introduction (i.e., more than 60 years ago), Google Trends was found to be a potentially useful tool for monitoring of disease outbreaks at the regional level in developed countries. These results show promising potential for Google Trends data to be used in real-time disease surveillance for many diseases, even in challenging contexts. The Pearson correlation coefficient was more suitable than Spearman’s rank correlation coefficient with respect to evaluating correlations between clinical case data and Google Trends search data.

## Data Availability

The datasets generated and/or analyzed during the current study are publicly available at: Monthly measles and rubella monitoring report, https://www.ecdc.europa.eu/en/rubella/surveillance-and-disease-data/monthly-measles-rubella-monitoring-reports Notified measles cases in japan, https://www.niid.go.jp/niid/en/measles-e.html Google Trends, https://trends.google.com/trends/ Global measles outbreaks, https://www.cdc.gov/globalhealth/measles/data/global-measles-outbreaks.html Survstat@rki 2.0, https://www.rki.de/EN/Content/infections/epidemiology/SurvStat/survstat_node.html.
